# Minor Salivary Gland Tumours of Upper Aerodigestive Tract: A Clinicopathological Study

**DOI:** 10.1155/2012/780453

**Published:** 2012-05-21

**Authors:** Grażyna Wyszyńska-Pawelec, Michał Gontarz, Jan Zapała, Mariusz Szuta

**Affiliations:** Department of Cranio-Maxillofacial Surgery of the Jagiellonian University, Rydygier Hospital, 1 Zlota Jesien Street, 31-826 Kraków, Poland

## Abstract

The aim of this retrospective study of 56 patients with minor salivary gland tumours (MSGTs) of the upper aerodigestive tract is to present demographic features, distribution of tumours as well as methods and results of treatment performed in our institution over a 10-year period. Of 221 patients with salivary gland tumours, 56 patients with MSGT were selected. There were 36 female and 20 male patients aged from 8 to 81 years. Male-to-female ratio was 1 : 2 in the group of benign MSGT and 1 : 1.7 in the group of malignant tumours. The palate was the most frequent site of MSGT (45.6%), followed by buccal mucosa (19.3%). Of all MSGTs 63.2% were malignant, and 36.8% were benign. Adenoid cystic carcinoma was the most common neoplasm (31.6%), followed by pleomorphic adenoma (29.8%). Surgery was the method of choice in the treatment of patients with MSGT. Postoperative defects were reconstructed by prosthetic obturators, local flaps, and free radial forearm flap. Relative survival for patients with malignant MSGT was 88% at three years and 71.5% at five years. MSGTs are more frequent in females and predominantly affect the palate. Malignant MSGTs are more common than benign.

## 1. Introduction

Primary tumours arising from minor salivary glands, distributed in the palate, buccal mucosa, tongue, lips, paranasal sinuses, pharynx, and larynx, are relatively uncommon. Minor salivary gland tumours (MSGTs) represent 9–25% of all salivary gland tumours [1] as well as 2-3% of all head and neck tumours [2] with a female preponderance [1–3]. Unlike major salivary gland neoplasms, the majority of MSGTs are malignant. The two most common malignant MSGTs are mucoepidermoid carcinoma and adenoid cystic carcinoma [3, 4]. Pleomorphic adenoma is the most frequent benign tumour arising from minor salivary glands. The palate, especially the junction between the soft and hard palate, is the most typical location of MSGTs [5].

Due to complex histopathology of MSGTs, diagnostics and treatment planning are challenging. Histological classification of salivary gland tumours is still changing (according to the latest WHO modification in 2005) [6].

 The aim of this retrospective study with MSGTs of the upper aerodigestive tract is to present methods and results of treatment performed in our institution over a 10-year period.

## 2. Material and Methods

 The records of 221 patients with salivary gland tumours were retrospectively reviewed, and the group of 56 patients with MSGTs, treated over a 10 year period, was selected.

Patients' demographic data, comorbidities, symptoms, tumour locations, diagnostic methods, histological diagnosis, and therapeutic methods, including reconstructive procedures, were analyzed. Radicality of surgery was assessed. Oncologic outcomes (recurrence and five year survival) were also analysed. Data was collected using Excel 2007 Microsoft Office.

## 3. Results

 Between 2000 and 2009, 56 patients with 57 MSGTs were treated in our department. The age ranged from 8 to 81 years. The mean age of the patients with benign MSGTs was lower (48.3 years) than of those with malignant MSGTs (51.3 years).

There were 36 females and 20 males with the male-to-female ratio of 1 : 2 in the group of benign MSGTs and 1 : 1.7 in the group of malignant tumours. The time either from occurrence of the first symptoms or from initial diagnosis to surgery, ranged from 2 months to 30 years (3 years and 2 months on average). Patients with benign MSGTs delayed the treatment for 4 years, whereas those with malignant MSGTs for 2 years and 8 months on average. The most common comorbidities in this study comprised of hypertension (13 cases), struma nodosa (5 cases), gastric ulcer (4), cholecystolithiasis (4) and less frequently chronic gastritis, diabetes, gout, and hypothyroidism. Tobacco smoking was declared by 13 (23.2%) patients.

In two cases, development of other salivary gland tumour preceded MSGTs. In the first patient, basal cell adenoma of the buccal mucosa followed adenoid cystic carcinoma of the maxilla. In the second case, acinic cell carcinoma of the hard palate followed pleomorphic adenoma of the parotid gland.

**Figure 1:**
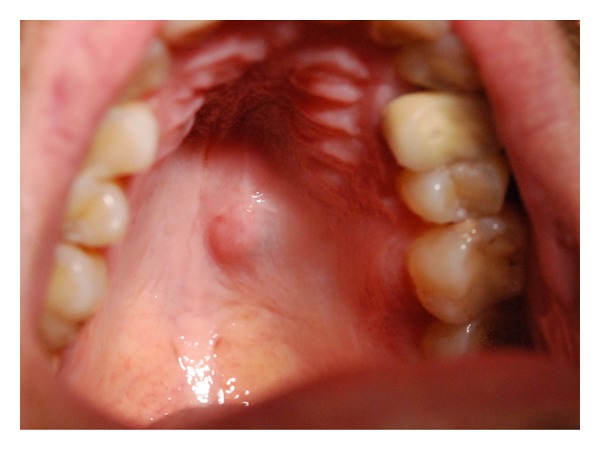
Adenoid cystic carcinoma of the hard palate.

**Figure 2:**
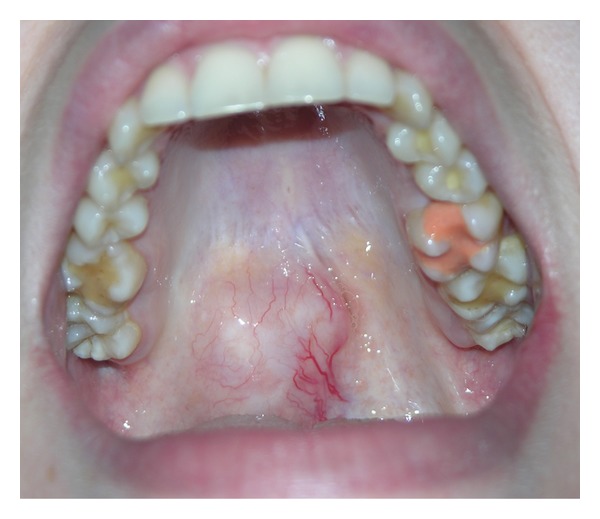
Pleomorphic adenoma of the junction between the soft and hard palate.

**Figure 3:**
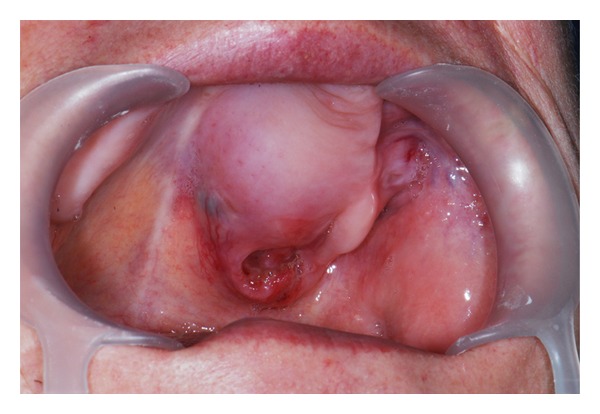
Adenoid cystic carcinoma of the left maxilla.

The majority of patients with MSGTs were asymptomatic, especially in the group of benign tumours, where pain was presented only in 4 cases. Patients with malignant MSGTs complained of pain (5 cases), bleeding ulceration (11 cases), and loose teeth. The palate was the most frequent site of MSGTs—26 cases (45.6%), followed by buccal mucosa and maxilla (Figures 1, 2, and 3). In addition, the palate was the most common site for both benign (53.8%) and malignant (46.2%) MSGTs. Majority of buccal mucosa tumours were benign (63.6%); however, MSGTs of maxilla, tongue, lips, floor of the mouth and retromolar region were all malignant. The location of benign and malignant MSGTs is depicted in Table 1.

Initial histologic diagnosis of MSGTs was obtained with open biopsy in 39 cases, fine needle aspiration cytology (FNAC) in 19 patients, and intraoperative frozen section analysis in 8. The results consistent with the final diagnosis were obtained in 7 FNAC examinations only, including 2 cases of malignant MSGTs. In two out of 14 open biopsies of benign MSGTs and in one out of 25 open biopsies of malignant tumours, the results were opposite to the final diagnosis. The results obtained by frozen section analysis showed negative resection margins in 8 cases. In 4 cases, preliminary histologic diagnosis was consistent with final examination.

 Histologic examination revealed 36 malignant MSGTs (63.2%) and 21 benign MSGTs (36.8%). Adenoid cystic carcinoma was the most common neoplasm: 18 (31.6%), followed by pleomorphic adenoma: 17 (29.8%). Of all malignant MSGT, mucoepidermoid carcinoma was the second most common neoplasm (14%).

Isolated cases of myoepithelioma, monomorphic adenoma, basal cell adenoma, and a hybrid tumour with features of both pleomorphic adenoma, and myoepithelioma were found in the benign group. Histologic types of MSGTs are presented in Table 2.

Stage of presentation was established in 34 cases out of 36 patients with malignant MSGTs. More than half of the patients presented in advanced stage disease (stage IV: 14, stage III: 5, stage II: 10, and stage I: 5 patients).

Surgery was the method of choice in the treatment of patients with MSGTs. Additional treatments in the malignant MSGTs group included neoadjuvant chemotherapy (1 case) and adjuvant radiotherapy (14 cases). Surgical treatment comprised of maxillectomy (various levels, including ethmoidectomy and exenteration of the orbit) in 18 cases and tumour excision in 18 patients, including resection of underlying bone in 7 cases. Concomitant neck dissection was performed in 9 patients. In this group, three patients required radical neck dissection, one selective neck dissection, and in 5 cases limited lymphadenectomy was performed.

**Figure 4:**
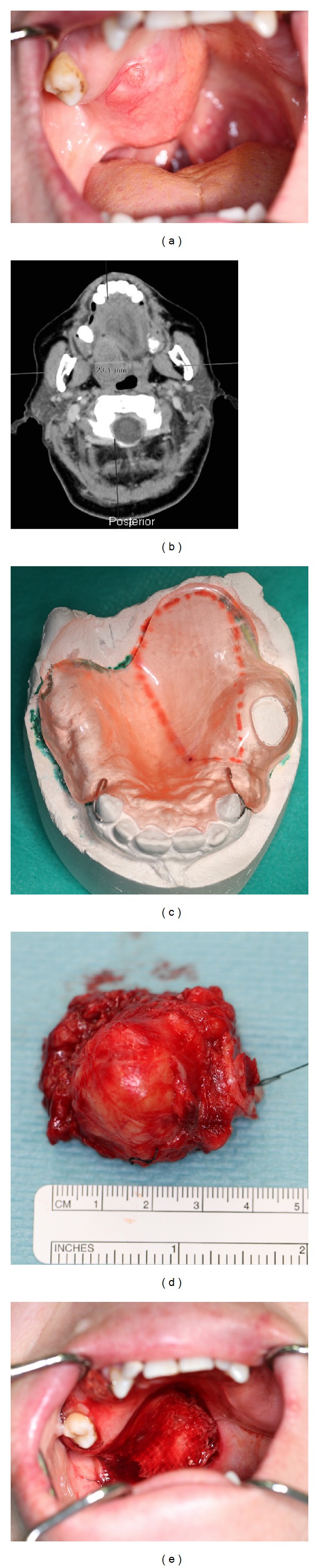
(a) Pleomorphic adenoma of the junction between the soft and hard palates in a 60-years-old female patient. (b) CT horizontal scan of the patient. (c) Prosthetic obturator. (d) Surgical specimen of resected pleomorphic adenoma. (e) Postoperative defect.

**Figure 5:**
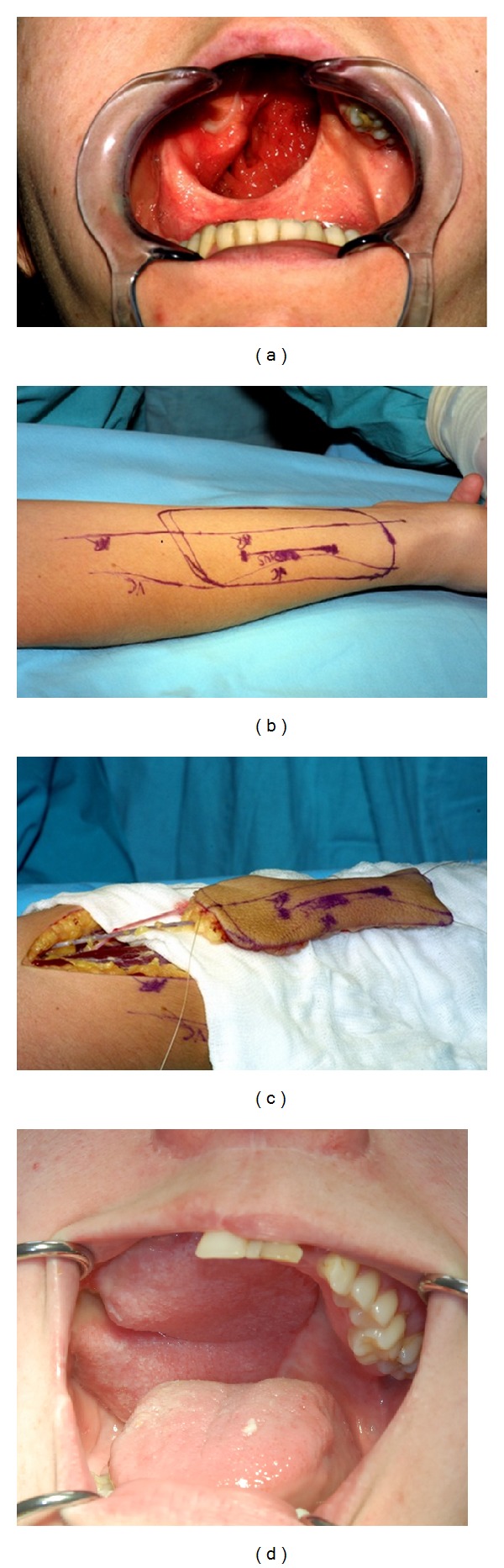
(a) Postoperative defect after partial maxillectomy due to low-grade mucoepidermoid carcinoma of the hard palate in 28-year-old female patient. (b) Radial forearm free flap designed. (c) Radial forearm free flap prepared for palatal reconstruction. (d) Result after 8 years following reconstruction of the palate by radial forearm free flap.

Postoperative defects were reconstructed by prosthetic obturators in 22 patients and local flaps in 10 cases (Figures 4(a)–4(e)). Additionally the cervical skin flap and buccal fat pad flap were used. Only in one case in this group, the defect was left to granulate. Delayed reconstruction was performed in 4 cases using palatal flaps, tongue flap, and free radial forearm flap (Figures 5(a)–5(d)). 

Patients with benign MSGTs required tumour excision in 15 cases, including bone resection in 7 cases, partial maxillectomy in 3 patients and tumour enucleation in 3. In this group, either simple wound closure or local flaps were used as well as prosthetic obturators.

Radical tumour excision was confirmed in 19 patients with malignant MSGTs and in 20 patients with benign neoplasms. Patients with positive margins had either wider resection or radiotherapy in malignant tumours, especially in cases where extension of surgical procedure was not possible. No recurrences were noted in patients with benign MSGTs. Nine patients with malignant tumours (8 adenoid cystic carcinoma and 1 adenocarcinoma) developed local recurrence which was observed 2 months to 11 years and 10 months (3 years and 11 months on average) following the primary surgical treatment. Secondary neck dissection was performed in 10 patients with clinically positive lymphadenopathy. Cervical metastases (levels IB, II, and III) were confirmed in 6 cases (16.2%) and occurred on average 11 months following the primary surgical procedure.

Three patients (8.3%) with adenoid cystic carcinoma developed distant metastases in lungs, liver, and bones, 2 to over 5 years following surgery, with a median time of 3 years and 6 months.

Relative survival of our patients with malignant MSGTs was 88% (25 cases) at three years and 71.5% (14 cases) at five years.

## 4. Discussion

This retrospective analysis concerned patients from the southern Poland, treated in the University Department of Cranio-Maxillofacial Surgery. The present study revealed that malignant MSGTs occurred in the older population than benign tumours (3 years on average). This was also reported by others [1, 2].

Review of the literature shows a female predilection among the MSGTs patients (male-to-female ratio ranging from 1 : 1.02 to 1 : 2) [1, 4]. Higher proportion of females was also found in our study (ratio of 1 : 1.8 for all MSGTs). Reported tendency of female predominance characteristic for benign MSGTs [7, 8] was also confirmed in the present series with a male-to-female ratio of 1 : 2. In patients with malignant MSGTs the ratio was 1 : 1.7.

Relatively slow and almost asymptomatic growth of MSGTs might not be noticed by a patient. Fortunately, these tumours are often found during routine dental examination [5].

Concomitant diseases of the digestive tract, such as gastric ulcer, chronic gastritis, and cholecystolithiasis were diagnosed in 10 (17.8%) patients in this series. However, we have not found studies indicating relation between diseases of the digestive tract and occurrence of MSGTs.

Tobacco smoking, irradiation, and history of prior neoplasms were mentioned in some studies [5, 9] as potential risk factors, although the causes of MSGTs are largely unknown. In the present study, 23.2% of patients smoked cigarettes. As far as prior neoplasms are concerned, two patients in our series developed two histologically different and independent salivary gland tumours.

According to other reports [1, 3, 8, 10], the palate was the most commonly affected site, and this was also seen in our study. In this location we found 45.6% of all MSGTs with predominance for benign tumours (53.8%). Moore et al. [5] suggests that MSGTs usually occurred at the junction of the hard and soft palates due to concentration of salivary glands in this region. However, the distribution of MSGTs found in the present study is different as the hard palate was the most common site in 47.6% of benign and 25% of malignant tumours.

Other anatomical sites of involvement differ in various studies. Pires et al. [3], Buchner et al. [10], and Yih et al. [4] have indicated the lips, whereas Venkata [7] indicated the alveolar mucosa as the second most common site. In the present study, the palate was followed by the buccal mucosa.

As in our study, review of the literature [10, 11] revealed that MSGTs affecting the retromolar region as well as the floor of the mouth were malignant. It is believed that malignant MSGTs predominate even up to 76% over benign tumours [5, 7, 8]. Greater proportion, 63.2% of malignant tumours, was also found in our series. On the other hand, the results reported in other studies [1, 3, 4, 10] showed lower incidence of malignant MSGTs.

In this study, adenoid cystic carcinoma was the most common pathological type of MSGTs (31.6%) followed by pleomorphic adenoma (29.8%), which was also a predominant benign MSGTs. Our findings were consistent with other series [1, 5, 10] as far as the frequency of pleomorphic adenoma was concerned but according to other reports [4, 10, 12], mucoepidermoid carcinoma was the most common malignant MSGTs. Studies concerning west European population [13–15] reported adenoid cystic carcinoma as the most common malignancy.

FNAC is recommended in the preoperative diagnosis of MSGTs, with 96% accuracy. Open biopsy of these tumours is controversial due to potential tumour seeding [5, 9, 16]. Frozen section analysis might lead to false-positive (1.1%) or false-negative (2.6%) rates [9]. In the present study, accuracy of these diagnostic tools was low.

In patients with MSGTs, computed tomography (CT) is a basic diagnostic examination in evaluation of bone destruction and infiltration of surrounding tissues. Especially extension into paranasal sinuses, nasal cavity, orbit, and pterygopalatine and infratemporal fossa can be appreciated. Detailed assessment of soft tissues involvement might be obtained by MRI imaging [5, 9].

The management of MSGTs remains primarily surgical including wide excision of malignant neoplasms and enucleation of encapsulated benign tumours. However, benign MSGTs of the palate, mainly pleomorphic adenomas, require excision with adequate soft tissue margin as well as removal, sometimes resection, of the underlying bone [5, 9].

Due to the low rate of cervical metastases in MSGT, elective neck dissections are not recommended [17, 18]. This rule was followed in the management of our patients.

Evaluation of postoperative quality of life of the patients with MSGTs depends on the method of reconstruction, especially in cases with palatal defects. Recently, primary closure of ablative defects by palatal, buccal and tongue flaps, temporalis myofascial flap, vascularised free flaps has been recommended [5, 19, 20]. Still used prosthetic obturators might be a reason for oronasal reflux, but they also enable direct visual control of the operated site, which is important in patients with malignant MSGTs. In available studies [9, 21, 22], five-year relative survival of the patients with MSGTs ranged from 50% to 89%, which was consistent with our findings.

## 5. Conclusions

1. relatively rare MSGTs are more frequent in females and predominantly affect the palate;
2. malignant MSGTs are more common than benign;
3. adenoid cystic carcinoma and pleomorphic adenoma are the most common MSGTs.

## Figures and Tables

**Table 1:** Tumour location.

Type of tumour	Location		Number of cases
Benign	Palate		14
	Hard palate	10
	Soft palate	1
	Hard + soft	3
	Buccal mucosa		7

Malignant	Palate		12
	Hard palate	9	
	Soft palate	3	
	Maxilla		11
	Buccal mucosa		4
	Labial mucosa		3
	Upper lip	2	
	Lower lip	1	
	Tongue		2
	Floor of the mouth		2
	Retromolar region		2

			Total: 57 tumours*

*In one patients two MSGTs were diagnosed.

**Table 2:** Histological type of minor salivary gland tumours.

Type of tumour		Number	
Benign		21	(36.8%)
Pleomorphic adenoma	17
Myoepithelioma	1
Pleomorphic adenoma and myoepithelioma	1
Monomorphic adenoma	1
Basal cell adenoma	1

Malignant		36	(63.2%)
Adenoid cystic carcinoma	18
Mucoepidermoid carcinoma	8
Adenocarcinoma	4
Acinic cell carcinoma	3
Papillary cystadenocarcinoma	1
Carcinoma ex pleomorphic adenoma	1
Undifferentiated carcinoma	1

		Total: 57 tumours*	

*In one patients two MSGTs were diagnosed.
